# Severity, Treatment, and Outcome of Acute Pancreatitis in Thailand: The First Comprehensive Review Using Revised Atlanta Classification

**DOI:** 10.1155/2017/3525349

**Published:** 2017-04-13

**Authors:** Supot Pongprasobchai, Peeradon Vibhatavata, Piyaporn Apisarnthanarak

**Affiliations:** ^1^Division of Gastroenterology, Department of Medicine, Faculty of Medicine Siriraj Hospital, Mahidol University, Bangkok, Thailand; ^2^Department of Medicine, Faculty of Medicine Siriraj Hospital, Mahidol University, Bangkok, Thailand; ^3^Division of Diagnostic Radiology, Department of Radiology, Faculty of Medicine Siriraj Hospital, Mahidol University, Bangkok, Thailand

## Abstract

*Background*. Severity and outcome of acute pancreatitis (AP) in Thailand are unknown. *Methods*. A retrospective study of 250 patients with AP during 2011–2014 was performed. Severity, treatment, and outcome were evaluated. Severity was classified by revised Atlanta classification. *Results*. The mean age was 58 years and 56% were men. Etiologies were gallstones (45%), alcohol (16%), postendoscopic retrograde cholangiopancreatography (14%), and idiopathic (15%). Overall, 72%, 16%, and 12% of patients had mild, moderately severe, and severe AP, respectively. Two major types of initial intravenous fluid were normal saline (64%) and Ringer's lactate solution (RLS, 28%). Enteral nutrition was given in 77% of patients with severe AP, median duration 48 hours, and via a nasogastric tube in 67% of patients. Necrotizing pancreatitis (NP) developed in 7% of patients, and 29% of them developed infection (median 17 days). The median length of stay was 6, 9, and 13 days, and the mortality rate was 1%, 3%, and 42% in mild, moderately severe, and severe AP, respectively. The overall mortality rate was 6%. *Conclusion*. The severity of AP in Thailand was mild, moderately severe, and severe in 72%, 16%, and 12% of patients, respectively. NP was not prevalent. Mortality was high in severe AP. Most treatments complied with standard guidelines except the underuse of RLS.

## 1. Introduction

Acute pancreatitis (AP) is an acute pancreatic inflammatory process triggered by abnormal activation of pancreatic enzymes and the release of several inflammatory mediators. The disease exhibits variable involvement of other regional tissues and remote organ systems [1]. The overall mortality rate of AP is approximately 5% [2]. There is a wide variation in the disease severity, ranging from mild, self-limiting disease that recovers within 3 to 5 days without mortality to severe, life-threatening disease with a mortality rate of 30% to 40% [3].

The Atlanta classification of 1992 provided a global consensus and universally applicable classification system for AP [1]. Although it is useful and widely accepted, some of the definitions used in this version of the classification remain confusing. A better understanding of the pathophysiology of organ failure (OF) and necrotizing pancreatitis (NP) and their outcomes, as well as improvements in diagnostic imaging techniques, has made it necessary to revise the Atlanta classification. The 2012 revision of the Atlanta classification includes clinical assessment of the severity of AP as mild, moderately severe, or severe and provides more objective terms with which to describe the local complications of AP. Thus, it is currently the gold standard for evaluating the severity of AP [4].

In Thailand, studies on AP are very scarce despite the importance of the disease [5–8]; this lack of data is partly due to the uncommonness of AP. Basic information on the severity and outcomes of AP in Thai patients using the current classification would represent a milestone in the development of a Thai guideline for AP; however, such data are lacking. In the present study, we evaluated the severity, treatment, and outcomes of patients with AP in our institution according to the 2012 revision of the Atlanta classification.

## 2. Materials and Methods

### 2.1. Study Design and Population

This retrospective study involved all adult patients who were admitted to Siriraj Hospital, Bangkok, Thailand, for the treatment of AP from December 2011 to December 2014. The study was approved by the Siriraj Institutional Review Board.

All adult patients aged ≥ 18 years who were diagnosed with AP by meeting two of the three diagnostic criteria for AP [abdominal pain, a serum amylase or lipase activity three times higher than the upper limit of normal, and pancreatitis documented by computed tomography (CT)] were enrolled [1, 4]. Only patients who initially presented to our hospital within 48 hours were included in the study. Patients with incomplete medical records, especially in the first 48 hours of disease, were excluded.

### 2.2. Clinical Data

Data regarding severity, treatment, and outcomes of AP were collected using designed case record forms.

### 2.3. CT Findings

All CT scans were reviewed by two of the authors: a gastroenterologist with expertise in pancreatic diseases (S.P.) and a specialized gastrointestinal radiologist (P.A.). The terminology used followed the 2012 revision of the Atlanta classification [4].

### 2.4. Definitions of Severity of AP and Local Complications

The severity of AP and terminology regarding systemic and local complications were classified according to the 2012 revision of the Atlanta classification [4].

### 2.5. Statistical Analysis

Descriptive statistics were used to summarize the patients' characteristics. Continuous data are presented as mean and standard deviation, whereas categorical data are presented as frequency and percentage. The chi-square test or Fisher's exact test was used to compare categorical variables among the three severity groups. A standard *t*-test or the Mann-Whitney *U* test was used to compare continuous variables. All statistical analyses were performed using SPSS software. A two-sided *p* value of <0.05 was considered statistically significant.

## 3. Results

### 3.1. Patients' Characteristics

The data of 272 consecutive patients with AP were reviewed. Twenty-two patients were excluded due to incomplete data. In total, 250 patients with AP met the eligibility criteria. One-hundred forty patients (56%) were male, and 110 patients (44%) were female. The mean age of the patients was 58 ± 17 years (56 ± 16 years in male and 62 ± 19 years in female), and 72% had various comorbidities (Table 1). Almost all patients (99%) presented with abdominal pain, 64% had referred pain to the back, and 1% presented with altered consciousness. The median duration of symptoms was 24 hours before admission, of which was not different between male (median 24 hours, range 6–48 hours) and female (median 24 hours, range 8–48 hours). Both the serum amylase and lipase levels had a sensitivity of 95% for a diagnosis of AP, and the combination of both enzymes was not superior to the use of either enzyme alone. One-fourth of the patients underwent CT at a median of 5 days after admission.

The etiologies of AP were gallstones (45%), alcohol-related disease (16%), postendoscopic retrograde cholangiopancreatography (ERCP) (14%), idiopathic (15%), and miscellaneous (10%). When comparing the etiology of AP in women to those in men, women had significantly more gallstone pancreatitis (60.9% versus 33.6%), but less alcoholic pancreatitis (4.5% versus 25.7%) (Table 2).

### 3.2. Severity of AP

**Figure 1:**
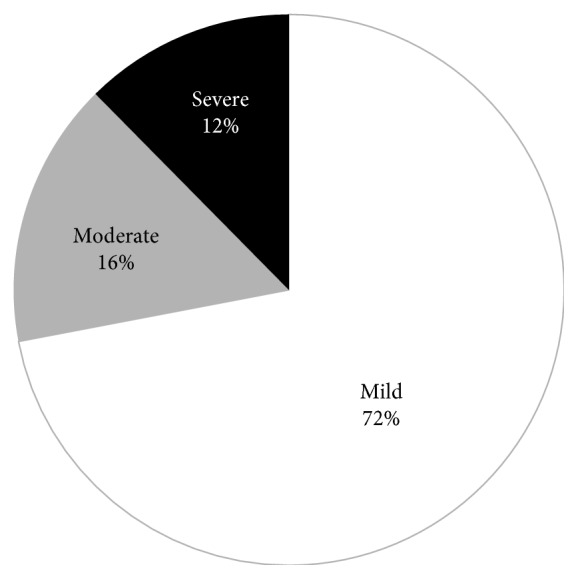
Severity of acute pancreatitis.

The severity of AP according to the 2012 revision of the Atlanta classification was mild, moderately severe, and severe in 72%, 16%, and 12% of patients, respectively (Figure 1). Eighty-two percent of patients had no OF, 6% had transient OF, and 12% had persistent OF.

### 3.3. Accuracy of Early Severity Assessment Tools to Predict Severe AP

Using the 2012 revision of the Atlanta classification as the gold standard, the sensitivity, specificity, positive predictive value, and negative predictive value of the two most commonly used severity assessment tools [bedside index for severity in AP (BISAP) score of ≥3 and the presence of systemic inflammatory response syndrome (SIRS) upon admission] are shown in Table 3. The Acute Physiology and Chronic Health Evaluation II (APACHE II) score was evaluated in very few patients; thus, this score was not analyzed.

### 3.4. Intravenous Fluid

**Figure 2:**
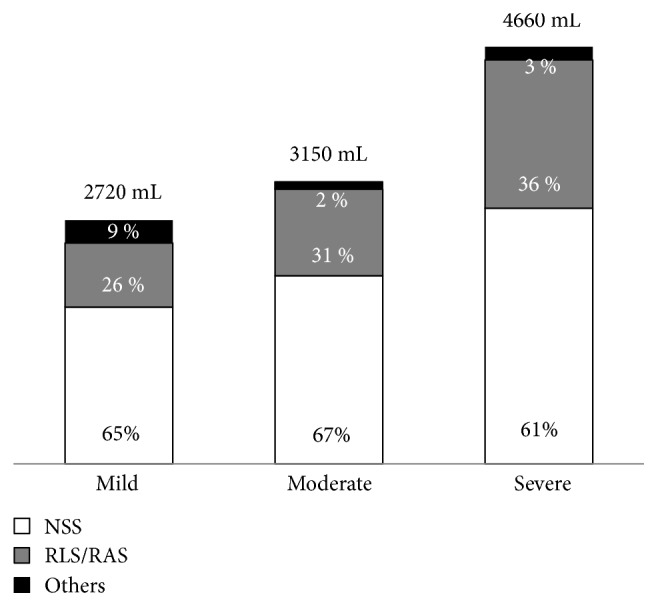
Types and amounts of intravenous fluid therapy during the first 24 hours. NSS, normal saline solution; RLS/RAS, Ringer's lactate solution/Ringer's acetate solution.

The two essential treatments of severe AP that are able to reduce OF and/or mortality are early fluid resuscitation and enteral feeding [9]. The mean total intravenous fluid resuscitation volume during the first 24 hours in the studied patients was 2720, 3150, and 4660 mL in patients with mild, moderately severe, and severe AP, respectively. The type of intravenous fluid was normal saline solution in approximately 60% of patients and Ringer's solution in approximately 30% (Figure 2).

### 3.5. Enteral Nutrition

**Figure 3:**
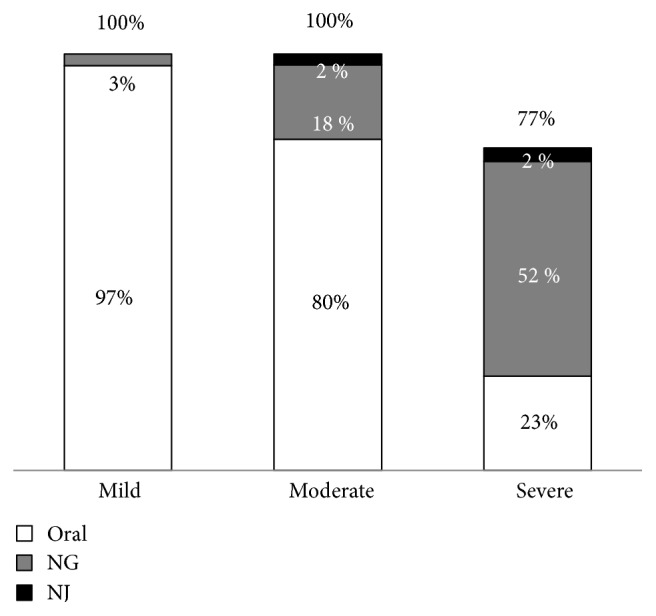
Routes of enteral nutrition. NG, nasogastric; NJ, nasojejunal.

All patients with mild and moderately severe AP and 77% of those with severe AP received enteral nutrition. The median time until the start of enteral feeding was 16, 24, and 48 hours in patients with mild, moderately severe, and severe AP, respectively. Among patients with mild AP, 97% received enteral nutrition orally and 3% required nasogastric feeding. Among patients with moderately severe AP, 80% received enteral nutrition orally, 18% received it via a nasogastric tube, and only 2% received it via a nasojejunal tube. In contrast, 52% of patients with severe AP required nasogastric feeding, 23% could take it orally, and 3% required nasojejunal feeding (Figure 3).

### 3.6. Necrotizing Pancreatitis and Local Complications

NP occurred in 17 patients (7%). Almost 70% of cases of NP involved parenchymal necrosis with or without peripancreatic necrosis, while only 30% involved solely peripancreatic necrosis. Regarding the local complications, 27 patients (11%) had acute peripancreatic fluid collections (APFC) and 13 patients (5%) developed acute necrotic collections (ANC). Pancreatic pseudocysts were detected in 2 out of 4 patients with APFC, who had follow-up CT. Walled-off necrosis (WON) occurred in 9 out of 12 patients with necrotizing pancreatitis or ANC, who had follow-up CT (Table 4).

### 3.7. Pancreatic Infection

Overall, 5 out of 17 patients (29%) with NP had infected necrosis. The diagnostic methods of infected necrosis were the presence of gas on CT (66.6%) and Gram staining or culture of specimens obtained from fine-needle aspiration or percutaneous drainage (50.0%). The median time until the diagnosis of pancreatic infection was 17 days (range, 7–25 days) after admission. All patients with pancreatic infections were treated by either percutaneous drainage (4 patients, 80%) or open necrosectomy (1 patient, 20%) (Table 5).

### 3.8. Outcomes

The median length of hospital stay was 6, 9, and 13 days in the mild, moderately severe, and severe AP groups, respectively, with significant differences (*p* < 0.001). Surgery was performed in two patients with severe AP. One patient underwent pancreatic necrosectomy, and another underwent exploratory laparotomy due to a diagnosis of generalized peritonitis and was found to have AP. No surgery was performed in the mild and moderately severe groups. The AP-related mortality rates were 1%, 3%, and 42% in the mild, moderately severe, and severe AP groups, respectively, with significant differences (*p* < 0.001) (Table 6). In the comparison of 13 patients with severe AP who died and 18 patients with severe AP who survived, there was no difference regarding the patients' age, gender, etiology of AP, timing of admission, the ICU care, type of intravenous fluid resuscitation, presence of local complications, and surgery (data not shown). The median ICU stays were 7 days and 8 days. The two deaths in the mild AP group had nonpancreatic causes (acute cholangitis with septicemia and upper gastrointestinal bleeding, resp.).

## 4. Discussion

The 2012 revision of the Atlanta classification is a recently proposed gold standard for the classification of AP [4]. The present study is among one of the few that have used the revised Atlanta classification to evaluate the severity, treatments, and outcomes of AP [10]. The results of this study are of importance and represent a milestone in the development of a guideline for AP in Thai patients. Our results highlight some important points regarding AP in Thai patients.

The age and sex of the patients with AP in this study were very similar to those of patients in recent studies from the West [11] and Japan [12]; however, the mean age was slightly higher than that in a study from China [13, 14]. The typical clinical presentation was abdominal pain in almost all patients. Referred pain to the back was present in two-thirds of the patients of the present study and in close to one-half of patients in another study [15].

The etiology of AP in this study was comparable with that in other studies from Asia [12, 13], with the leading causes of AP being gallstones and alcohol-related disease. However, gallstones were more prevalent in female, while alcohol was so in male, of which were well known. The incidence of gallstone pancreatitis was not different from that in our previous study in 2007 [7]; however, the incidence of alcohol-related AP was significantly lower with a paradoxical increase in post-ERCP pancreatitis, which was the fourth most common etiology in the present study. This can be explained by the increasing numbers of ERCP procedures performed during the last decade in our institution, which is a tertiary hospital. Idiopathic AP was the third most common etiology in the present study; it fell within the acceptable range (≤20%) suggested by most guidelines [9, 16–19].

The present study demonstrated that the severity of AP in our institution according to the 2012 revision of the Atlanta classification was mild, moderately severe, and severe in 72%, 16%, and 12% of patients, respectively. These data are consistent with recent data from Western studies, which reported mild AP in 70% to 85% of patients, moderately severe AP in 10% to 15% of patients, and severe AP in 5% to 15% of patients [20, 21]. Very few data are available from Asia because most studies were published before the endorsement of the 2012 revision of the Atlanta classification [12]. However, a recent study from China [22] that utilized the 2012 revision of the Atlanta classification reported mild, moderate, and severe AP in 30%, 53%, and 17% of patients, respectively. The major difference between that study and ours occurred among the patients with moderately severe AP; the incidence of both OF and local complications differed considerably between these two studies. The reason for the very high prevalence of moderately severe AP in this Chinese study is unclear, but we feel that it is too high when compared with those in the other studies from Western countries [20, 21]. It is possible that the Chinese study did not exclude transferred patients, and therefore, the rates of moderate and severe AP were higher. Our study had a strength that we excluded transferred patients and, therefore, would represent the more accurate picture of the severity of AP. Another possible reason for the lower prevalence of moderately severe AP in our study might be the lack of CT examination in all patients. Our CT examinations were mainly performed in patients suspected to have severe AP, as reflected by the 25% frequency of CT in our study. Some patients with mild AP might have actually had local complications and would have been classified as having moderately severe AP if CT had been performed. However, our practice of performing CT complied well with all standard guidelines of AP [9, 19, 23], which recommend CT mainly for patients with clinically severe AP.

The use of early severity assessment tools for AP is another interesting topic. A recent meta-analysis [24] and a comprehensive review by the present authors [25] concluded that the four best tools with which to predict the severity of AP within the first 24 hours were the presence of SIRS and the APACHE II, BISAP, and Japanese severity scale scores upon admission. All of these parameters have an advantage of a very high negative predictive value with which to rule out severe AP [24, 25]. In our institute, the presence of SIRS and the BISAP score are frequently used, whereas the APACHE II score and the Japanese severity scale score are rarely used. The present study confirmed that the presence of SIRS and the BISAP score on admission did very well in ruling out severe AP due to their high NPV (94%–97%). The presence of SIRS on admission was slightly more effective than the BISAP score in excluding severe AP.

Fluid resuscitation during the first 24 hours and enteral nutrition are currently the two most critical and essential treatments of AP because they can reduce both OF and mortality [26]. In the present study, the intravenous fluid volume administered to patients with severe AP complied well with the guidelines, which recommend administration of 3 to 5 L of fluid during the first 24 hours [9, 19, 23]. However, we found that instead of using Ringer's solution, which is preferred by most guidelines [9, 19, 23], most of our patients received normal saline solution. The reason for this difference is unknown, but we hypothesized that it might be related to a lack of knowledge among physicians or a more familiarity with normal saline solution than Ringer's solution. This problem is critical and must be urgently solved systematically by providing education to the physicians or making a clinical institute policy. Conversely, enteral nutrition, which is the most effective treatment for AP and is endorsed by all guidelines [9, 19, 23], was prescribed to almost all patients in the present study.

Regarding necrotizing pancreatitis and local complications of AP, an interesting finding was that NP was not prevalent. The 7% frequency of NP in the present study was much lower than the 10% to 20% frequency in most studies. The exact reason for this difference is unknown. However, we hypothesize that it might be due to the undiagnosed pancreatic necrosis, particularly in patients with clinically mild AP, because CT usually was not done in such patients. Further study to determine the presence of moderately severe AP (from local complications) in patients with clinically mild AP in our country is required. Another possible reason could be the misclassification of some ANC as APFC, particularly during the first week of the disease because majority of the CT in the present study were performed within the first week. It is also the possible reason for the very low incidence of late local complications of AP, pseudocyst, and WON in the present study because we did not have a systematic follow-up CT for patients who had APFC and ANC. Therefore, we might underdiagnose many pseudocyst and WON.

The major outcomes of AP in the present study were similar to those in most guidelines [2]. The median hospital stay in our study was 6 days (6, 9, and 13 days for mild, moderate, and severe AP, respectively), which is similar to that reported in other studies [22]. Surgery was rare and was only performed in 2 of 250 patients. The mortality of AP in the present study was within the range reported in standard guidelines [2] and another recent study [22]. However, the 42% mortality rate of severe AP in this study was slightly higher than it should be (20–30%) [2]. We could not find any reasonable explanation for this high mortality, including the timing of admission, patients' age, ICU admission, type of intravenous fluid, enteral feeding, presence of local complications, or surgery. Finally, we could not deny that it might be due to the quality of our intensive care. Thus, this is another opportunity for improvement. Normally, mild AP should have a mortality rate near 0%. In the present study, however, two patients (1%) with mild AP died. The causes of death were cholangitis with severe *Pseudomonas aeruginosa* septicemia and upper gastrointestinal bleeding, respectively, neither of which was directly caused by AP.

The strength of the present study is that it is the first comprehensive study on AP in Thai patients. The “gold standard” 2012 revision of the Atlanta classification was used. All CT scans were reviewed by experts in pancreatic disease according to the 2012 revision of the Atlanta classification, and all treatments aligned with all recent guidelines with no limitations of the facilities. However, the weaknesses of this study are the retrospective and observational nature of the study and the study was conducted in a university hospital setting; therefore, the results might not accurately represent the clinical picture of AP in Thailand. Additionally, the etiology of AP was post-ERCP in 14% of the patients in this study, which would not be found in the community setting. Nevertheless, when we calculated the severity of AP after excluding the 35 patients with post-ERCP pancreatitis, the results were unchanged (data not shown).

## 5. Conclusions

The severity of AP in our study was mild, moderately severe, and severe in 72%, 16%, and 12% of patients, respectively. NP was not prevalent, and infected necrosis occurred in one-third of NP. Mortality was high in severe AP. Most treatments of AP, particularly the amount of intravenous fluid administration and enteral feeding, complied well with the standard guidelines except the use of normal saline solution rather than Ringer's solution. Further strategies to improve the intensive care quality of severe AP and to endorse the use of Ringer's solution are urgently needed. Prospective multicenter data collection is also advocated.

## Conflicts of Interest

The authors declare that there is no conflict of interests regarding the publication of this paper.

## Figures and Tables

**Table 1:** Characteristics of the 250 patients with acute pancreatitis.

Age in years	58 ± 17
Male	140 (56)
Comorbidities	180 (72)
Hypertension	117 (47)
Diabetes	64 (26)
Dyslipidemia	35 (14)
Malignancy	29 (12)
Cardiovascular disease	28 (11)
Chronic kidney disease	16 (6)
Chronic obstructive pulmonary disease	5 (2)
HIV/AIDS	3 (1)
Department of admission	
Medicine	173 (69)
Surgery	77 (31)
Signs and symptoms	
Abdominal pain	247 (99)
Back pain	160 (64)
Altered consciousness	2 (1)
Dyspnea	1 (0.4)
Duration of symptoms in hours	24 (7–48)
Serum pancreatic enzymes	
Amylase ≥ 3 times upper limit of normal	229 (95)
Lipase ≥ 3 times upper limit of normal	212 (95)
Amylase and lipase ≥ 3 times upper limits of normal	196 (91)
Performance of CT	62 (25)
Number of days after admission	5 (1–39)
Etiology of AP	
Gallstones	113 (45)
Alcohol-related disease	41 (16)
Post-ERCP	35 (14)
Miscellaneous	24 (10)
Idiopathic	37 (15)

Age and BMI are presented as mean ± standard deviation; all other data are presented as *n* (%) or median (range). AP: acute pancreatitis; CT: computed tomography; ERCP: endoscopic retrograde cholangiopancreatography.

**Table 2:** Etiology of acute pancreatitis according to the gender.

Etiology, *n* (%)	Male (*n* = 140)	Female (*n* = 110)	*p* value
Gallstone	47 (33.6)	67 (60.9)	<0.001
Alcohol	36 (25.7)	5 (4.5)	<0.001
Post-ERCP	19 (13.6)	16 (14.5)	0.823
Idiopathic	23 (16.4)	14 (12.7)	0.413

All data are presented as *n* (%). ERCP: endoscopic retrograde cholangiopancreatography.

**Table 3:** Accuracy of the two most common methods to predict severe acute pancreatitis.

	Sensitivity (%)	Specificity (%)	PPV (%)	NPV (%)
SIRS upon admission	87	69	29	97
BISAP score of ≥3	61	90	46	94

SIRS: systemic inflammatory response syndrome; BISAP: bedside index for severity in acute pancreatitis; PPV: positive predictive value; NPV: negative predictive value.

**Table 4:** Necrotizing pancreatitis and local complications of acute pancreatitis.

*Necrotizing pancreatitis*	
All	17 (7)
Parenchymal necrosis	3 (1)
Peripancreatic necrosis	5 (2)
Both	9 (4)

*Local complications*	
Acute peripancreatic fluid collection	27 (11)
Acute necrotic collection	13 (5)
Pancreatic pseudocysts	2 (1)
Walled-off necrosis	9 (4)

Data are presented as *n* (%).

**Table 5:** Pancreatic infection status of the 17 patients with necrotizing pancreatitis and their treatments.

Necrotizing pancreatitis	
Sterile	12 (71)
Infected	5 (29)
Percutaneous drainage	4
Surgery	1

Data are presented as *n* (%).

**Table 6:** Treatment outcomes.

	Mild (*n* = 179)	Moderately severe (*n* = 40)	Severe (*n* = 31)	*p*
Length of stay in days	6 (1–29)	9 (3–147)	13 (1–110)	<0.001
Surgery	0 (0)	0 (0)	2 (100)	0.015
Mortality	2 (1)	1 (3)	13 (42)	<0.001

Data are presented as median (range) or *n* (%).
